# Unboxing Deep Learning Model of Food Delivery Service Reviews Using Explainable Artificial Intelligence (XAI) Technique

**DOI:** 10.3390/foods11142019

**Published:** 2022-07-08

**Authors:** Anirban Adak, Biswajeet Pradhan, Nagesh Shukla, Abdullah Alamri

**Affiliations:** 1 Centre for Advanced Modelling and Geospatial Information Systems (CAMGIS), Faculty of Engineering & IT, School of Civil and Environmental Engineering, University of Technology Sydney, Sydney, NSW 2007, Australia; anirban.adak@student.uts.edu.au (A.A.); nagesh.shukla@uts.edu.au (N.S.); 2 Earth Observation Centre, Institute of Climate Change, University Kebangsaan Malaysia, UKM, Bangi 43600, Selangor, Malaysia; 3 Department of Geology and Geophysics, College of Science, King Saud University, Riyadh 11451, United Arab Emirates; amsamri@ksu.edu.sa

**Keywords:** sentiment analysis, food delivery service, deep learning, explainable AI, LIME, SHapley

## Abstract

The demand for food delivery services (FDSs) during the COVID-19 crisis has been fuelled by consumers who prefer to order meals online and have it delivered to their door than to wait at a restaurant. Since many restaurants moved online and joined FDSs such as Uber Eats, Menulog, and Deliveroo, customer reviews on internet platforms have become a valuable source of information about a company’s performance. FDS organisations strive to collect customer complaints and effectively utilise the information to identify improvements needed to enhance customer satisfaction. However, only a few customer opinions are addressed because of the large amount of customer feedback data and lack of customer service consultants. Organisations can use artificial intelligence (AI) instead of relying on customer service experts and find solutions on their own to save money as opposed to reading each review. Based on the literature, deep learning (DL) methods have shown remarkable results in obtaining better accuracy when working with large datasets in other domains, but lack explainability in their model. Rapid research on explainable AI (XAI) to explain predictions made by opaque models looks promising but remains to be explored in the FDS domain. This study conducted a sentiment analysis by comparing simple and hybrid DL techniques (LSTM, Bi-LSTM, Bi-GRU-LSTM-CNN) in the FDS domain and explained the predictions using SHapley Additive exPlanations (SHAP) and Local Interpretable Model-Agnostic Explanations (LIME). The DL models were trained and tested on the customer review dataset extracted from the ProductReview website. Results showed that the LSTM, Bi-LSTM and Bi-GRU-LSTM-CNN models achieved an accuracy of 96.07%, 95.85% and 96.33%, respectively. The model should exhibit fewer false negatives because FDS organisations aim to identify and address each and every customer complaint. The LSTM model was chosen over the other two DL models, Bi-LSTM and Bi-GRU-LSTM-CNN, due to its lower rate of false negatives. XAI techniques, such as SHAP and LIME, revealed the feature contribution of the words used towards positive and negative sentiments, which were used to validate the model.

## 1. Introduction

COVID-19 lockdowns and quarantines have increased the demand for online food delivery service (FDS) organisations, such as Uber Eats, Deliveroo and Menulog, because restaurants were instructed to stop providing dining services [1,2]. According to the research of Morgan Stanley [3], the restaurant sector has undergone significant changes as a result of COVID-19 and social distancing. They further added that the online delivery penetration share in the market might have moved ahead 2 to 3 years due to COVID-19. Furthermore, the Roy Morgan Research report [4] suggests that the nationwide lockdown in the middle of 2020 and an extended lockdown after that “supercharged” the rise of meal delivery services such as Uber Eats, Menulog, HelloFresh, Deliveroo, and DoorDash. The young generation, especially customers up to 40 years old, are ordering more while working from home [4]. Due to the development of online food delivery markets during the COVID-19 pandemic, FDSs offer a diversity and a variety of eateries to the convenience and comfort of homes and businesses. New cuisines have been brought into the country due to increased immigration from other countries [5]. Customers have access to a variety of meal selections and the possibility of ordering from the greatest diners and restaurants in town from the comfort of their own home or workplace. Due to the widespread usage of smartphone apps and the accessibility of the global positioning system, delivering food to a customer’s precise location is no longer a problem [6]. Customers may track the status of their orders starting from the time they place it. The competition between FDS organisations is getting tougher; now, Doordash which already holds a significant market share for FDS in the US, wants to enter Australia and encroach on their competitors’ share and grow bigger [7]. Additionally, Menulog is investing into advertisements by bringing Katy Perry into their campaigns to compete with Uber Eats [8]. Uber Eats, Deliveroo and Menulog [9] are global ordering and delivery marketplace systems that rely on a cost-intensive business model but handle all delivery logistics. These organisations operate on a commission basis and offer restaurant and food business owners a complete sales solution at no additional cost. Applications for FDS allow users to place orders, order food from restaurants, and have it delivered to them with just a few phone taps. Customers can choose from a variety of meal alternatives at a chain of restaurants. Such services are in high demand, thereby making online food providers happy. With the increase in orders and comments, most businesses are looking for ways to better utilise the data to identify areas where they can improve customer satisfaction. Despite their large sales and investments, FDS organisations continue to struggle with profitability due to high operating costs. Predatory pricing is a common strategy used by businesses to beat the competition by drastically reducing meal costs [10]. As online FDSs rely largely on restaurants, they have little control over the quality of the food. If a customer is dissatisfied with a meal or service, then the FDS organisation is responsible for the revenue loss. Thus, businesses, such as Sprig [11] and Munchery [12], have been forced to close due to a lack of revenue.

Customers primarily look for reviews and recommendations of others when they order cuisines from online FDSs. Positive reviews drive customers to make a decision on ordering food from one restaurant, whereas negative reviews help them look for other options [13]. FDS companies can look for the negative comments towards common complaint types, such as customer service, food quality, cost and slow delivery service, to understand the improvement areas to enhance customer satisfaction. A review or feedback system is now integrated into the portals or social media platforms of restaurants and FDSs. However, due to the overwhelming volume of review data dispersed over numerous platforms and the dearth of customer service experts needed to examine and respond to each of these comments, only a few companies actually respond to consumer feedback [14]. Organisations no longer need to hire customer service experts to read each review because artificial intelligence (AI) can help them in solving problems and saving money [15,16].

Sentiment analysis is an automated process used to determine the emotions and sentiments of customers towards the food or service [17]. Machine learning (ML) and deep learning (DL) techniques can be used to perform sentiment analysis. Researchers have recently focused on DL, taking inspiration from DL results in other domains, such as computer vision [18], medical image analysis [19], speech recognition [20], and natural language processing (NLP) [21]. Although the accuracy of DL models is higher compared with ML techniques, they lack the explainability of the black box model [22]. A neural network with layers of interconnected nodes, such as an input layer, multiple hidden layers, and an output layer, is known as DL [23]. DL classifiers attempt to mimic the human brain by making decisions by taking raw data, extracting features, and adjusting weights and bias [24]. Each layer builds on the inputs from the previous layers and passes them to the next layer, a process known as forward propagation [23]. The prediction error is calculated by using a loss function, such as gradient descent, and the error is corrected by adjusting the weights and bias of the nodes by moving backwards in time, a process known as backpropagation [23]. The DL model predicts the output by using forward and backpropagation processes, correcting errors and weights until it achieves optimal prediction. Nonlinearities, autoextraction of features from raw data, dynamic weight adjustments between nodes for error correction, and the reliance on how strong the input weights are to establish the connection with the nodes in the next layer make it difficult to visualise or interpret the model in terms of the reasoning behind DL classifier decisions. Researchers have started developing posthoc methods to explain the decisions made by DL classifiers, such as SHapley Additive exPlanations (SHAP) [25] and Local Interpretable Model-Agnostic Explanations (LIME) [26], and apply them in some domains, such as spatial drought forecasting [27].

## 2. Related Work

Over the past couple of decades, there has been a good amount of research published on the application of ML techniques to perform sentiment analysis in the FDS domain [13,28,29,30,31]. Sentiment analysis of customer reviews from tweets for various FDSs, such as Swiggy, Zomato and Uber Eats, was performed to understand consumer satisfaction [32]. The customer reviews were pulled from Twitter using R-Studio, and the Lexicon-based sentiment analysis method was used on the tweets. The tweets were analysed and further used to provide feedback and recommendations to the business. Another study compared different machine learning techniques such as Decision Tree (DT), Naïve Bayes (NB), Logistic Regression (LR), and Support Vector Machine (SVM) [31] to analyse and classify customer sentiment. The DT model achieved an accuracy of 89%, NB achieved 82.5%, LR achieved 90%, and SVM achieved 91%. Regarding the performance of the models in terms of computing time, the DT model required 1 h 4 m 32 s to train whereas SVM required 6320 ms. In the experiment, six year-wise datasets were used from 2015 to 2020, and it was found that the accuracy of SVM for the 2015 dataset was 89%, for the 2016 dataset it was 92%, and for the 2020 dataset it was 92% [31], which suggests that it will be beneficial to integrate the solution with other applications which can be useful to understand the customer feelings towards different products and services. The findings of their study indicate that the food and beverage industry can use the ML model to attract and retain customers by handling customer complaints.

In another significant work, Noor [33] compared the results of Lexicon, SVM, Natural Language Processing (NLP) and Text Mining from different works and found that Lexicon achieved the highest accuracy of 87.33% compared to other methods. However, it would be difficult to perform a sentiment analysis in languages other than English [13]. Additionally, domain adaptation must be taken into account while creating models because a word in one domain may have a different meaning in another. For example, ‘lightweight’ is a positive sentiment word for the electronics domain, whereas it is a negative sentiment word for kitchen appliances [13]. ML/DL techniques can overcome the challenge of domain adaptation by training the model from the same domain dataset. There may be several words used for the same aspect in customer reviews. For instance, the terms “LCD” and “screen” refer to the same thing in the context of a mobile phone. In the context of movies, pictures and movies are synonymous, but they are not in the context of cameras, where they refer to two different things. Furthermore, the terms “photo” and “picture” are synonymous in the camera industry [13]. Traditional dictionary-based lexicon training approaches do not work well as they are strictly limited to a smaller number of words, whereas ML/DL techniques can overcome these problems [34].

During the COVID-19 pandemic, many businesses received “1 star” reviews for being closed during lockdown. FDS Yelp received poor reviews for slow service or heat waves in the seating areas. The restaurants need to know about their customers’ complaints and expectations, which can be found through sentiment analysis [28]. Although conventional ML techniques have performed well in analysing online review data, they were limited in processing natural data in a raw format [30]. However, the Deep Learning (DL) technique solves this problem through its computational model which involves multiple processing layers to automatically discover the word pattern from a vast amount of data [35]. Several researchers have implemented deep learning to analyse customer sentiments in their domain [36,37,38,39,40]. A recent work [28] built and compared two ML and DL models to perform sentiment analysis on reviews extracted from the Yelp website. For the traditional models, the Gradient Boosting Decision Tree (GBDT) and the Random Forest classifier were applied whereas in terms of DL models, Simple Embedding + Average Polling and Bidirectional LSTM (Bi-LSTM) classifiers were used. The study found that the DL technique Bi-LSTM was more effective in generating subtopics whereas Simple Embedding + Average pooling performed better in customer review prediction tasks. The study had limitations in terms of the DL model, in that although it showed higher accuracy over ML models, it was criticised for being black box-based and uninterpretable in nature [28]. Thus, in this study, it was found that DL models performed better than ML models in terms of accuracy, but lack interpretability which results in a lack of trust in terms of its usage.

According to Nurdin [41], DL in NLP tasks, especially for sentiment analysis, has achieved remarkable progress due to the availability of a large amount of data. The XAI method must be used in conjunction with DL models to provide details about what drives the model to predict outcomes. The author analysed the DL models by using XAI methods, such as LIME, SHAP and Anchor. Present research [42] suggests that XAI methods, such as SHAP and LIME work on time-series data. In the medical field, sentiment analysis helped to understand the emotions and opinions of the patients by using DL models. However, DL models have the drawback of not being human interpretable, thereby raising concerns about the model’s interpretability. Another research work in the medical field [43] revealed that few studies have been performed to explain the decision-making process and actions of DL models. Ref. [44] expressed the need to uncover the ML models by using XAI, which is utilised for the sentiment analysis of hotel guest reviews. XAI techniques are recommended for examining DL models in other industries. However, to the best of our knowledge, no evidence was found on the application of XAI techniques on DL models in the FDS industry to analyse customer reviews.

The research aims of this study were to address the gaps identified in the literature by answering the following research questions:

- Which DL classifier will be best suited to pick FDS customer complaints from feedback and work on its solution?
- Can XAI techniques, such as LIME or SHAP, provide explanations for sentiment prediction and help to build trust in the DL model created from the previous question?

The main contributions of this study that differentiate it from similar studies are as follows:

Contribution I: This study compared the DL techniques and selected the best DL classifier suitable for the FDS domain to predict the negative sentiments from customer feedback that can be further used to improve customer satisfaction.

Contribution II: The prepared DL model was tested with XAI techniques to validate the model’s logic for prediction and build trust for the organisations and industries who use it.

The novelty and main contribution of this research work come from building a DL model and then explaining it using the XAI technique to validate the model’s logic for food industry application. On the basis of the recommendations and gaps found in a recently published review paper [13], we considered three different types of DL techniques, namely, LSTM, Bi-LSTM and Bi-GRU-LSTM-CNN, to be used on the customer review data extracted from the ProductReview website. LSTM and Bi-LSTM are forms of RNNs, which are fit for temporal data that are in a sequence. CNN is mostly used in spatial data, such as images, and GRU is a unit that is similar to an LSTM unit but does not have an output gate. We created a hybrid model (Bi-GRU-LSTM-CNN) with a combination of BiLSTM, GRU and CNN to perform sentiment analysis in the FDS domain.

Product Review (https://www.productreview.com.au, accessed on 1 June 2022) is an Australian customer review website that stores the customer reviews for multiple brands across various products and services. Six FDS organisations, such as Uber Eats, Menulog, Youfoodz, Deliveroo, My Muscle Chef and Macros, were selected from the ProductReview website for their customer review data. A DL model was built on 13,621 customer reviews pulled from the selected FDS organisations. The accuracy of the DL models was compared by using a confusion matrix, and then black box DL models were interpreted by using XAI techniques, such as SHAP and LIME. The contribution information of every word (feature) in a customer review sentence using SHAP and LIME techniques was utilised to analyse the outcome of the DL model.

The rest of this paper is organised as follows: Section 2 describes the methodology, including the data collection, data splitting, data cleaning and preprocessing of the DL methods (LSTM, Bi-LSTM and Bi-GRU-LSTM-CNN) and XAI techniques, such as SHAP and LIME. Section 3 presents the results. Section 4 discusses the results and offers a viewpoint. Section 5 provides the key findings and future research directions.

## 3. Methodology

The goal of the research is to develop highly accurate DL models and compare them to pick the best for performing sentiment analysis in the FDS domain. As stated in the literature review, previous work [31] in the FDS domain has shown DL models achieving higher accuracy than ML models and hence this research also focussed on DL models to attain higher accuracy to predict customer sentiments from reviews. Additionally, it was observed that the application of DL models in the FDS domain [29,31] causes; therefore, interpretability issues, Explainable Artificial Intelligence (XAI) techniques SHapley Additive Explanations (SHAP) and Local Interpretable Model-agnostic Explanations (LIME) were implemented to overcome the problem of interpretability of the black box DL models. There are several XAI techniques researched so far in other domains [27,45,46,47], but to the best of our knowledge, none have been applied in the FDS domain. The scientific contribution of this research is in combining the DL model along with the XAI technique as a package solution in the FDS domain to attain high accuracy and explainability to perform sentiment analysis on customer reviews.

**Figure 1:**
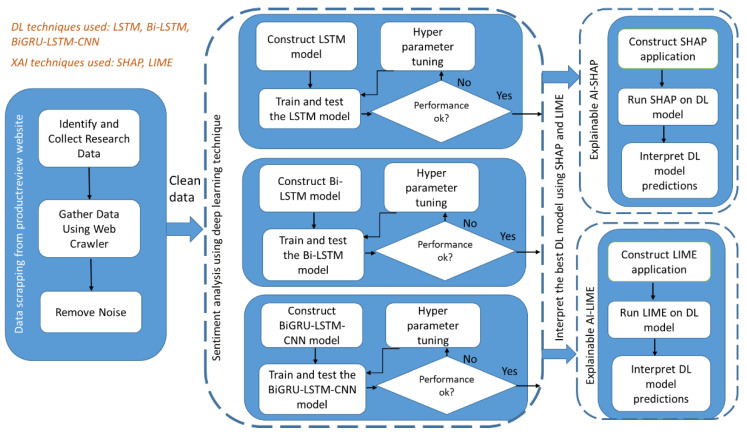
Methodology flow chart adopted in this work.

The methodology was designed by keeping the following two goals in mind: (a) perform sentiment analysis using DL models and compare, and (b) explain the predictions by showing the most significant features that contribute to a customer sentiment. The design of the research development methodology that was used to conduct the research presented in this study is shown in Figure 1. The methodology has three major parts–part 1 focused on data scrapping from the ProductReview website; part 2 which focused on developing different DL models (LSTM, Bi-LSTM, Bi-GRU-LSTM-CNN) and assessed the model’s performance; part 3 which focused on explaining the predictions made by the best suitable DL model using XAI techniques (SHAP and LIME).

The ProductReview dataset was split into test and training datasets. Three different DL models (LSTM, Bi-LSTM, Bi-GRU-LSTM-CNN) were developed, trained and tested on the dataset. Several rounds of testing and fine-tuning of the hyper parameters were performed to finalise the DL model’s architecture and then fine-tune its performance. The DL models were compared in terms of their accuracy and other key parameters for the FDS domain. The best DL model was picked after comparison, and XAI techniques (SHAP and LIME) were used to interpret the DL model. The explanations were provided, thereby obtaining the word features that contributed to positive or negative sentiments. The sections that follow provide more information.

### 3.1. Data Collection

Productreview.com.au is an Australian website that gathers consumer feedback on a variety of products and services. Overall, 13,621 customer reviews were collected from various FDS companies, such as Uber Eats, Menulog, Youfoodz, Deliveroo, My Muscle Chef and Macros, from the ProductReview website via web scraping. The below dataset example (Table 1) shows the various attributes present along with customer reviews. Review comments and star ratings were used to train the DL models.

### 3.2. Data Splitting

**Figure 2:**
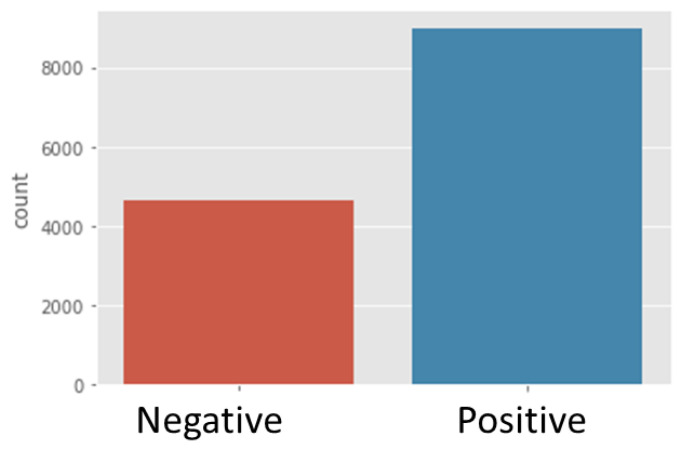
Negative and positive sentiment count.

The dataset was grouped into the following binary sentiment tasks: positive and negative classes. The positive class was labelled as ratings of 4 and above, and the negative class was labelled as a rating of 2 or below. The dataset was then divided into 8995 positive reviews and 4626 negative reviews (Figure 2). Rating 3 was not placed in any of the classes.

### 3.3. Data Cleaning and Preprocessing

The labelled customer review data were cleaned by reducing the noise and normalising each word to lowercase. Further punctuation, such as question marks, commas, colons, hash signs and website URLs, were removed to reduce the noise of the data. Some review data sequences were truncated or padded to provide a fixed length and mark all the sequence data into a standard length. For the training data, one of the requirements for LSTM models is to have a fixed length for the input sentence length of the review data. We set the customer review data length to 100.

### 3.4. Design

- In Study 1, a sentiment analysis using DL techniques was performed, such as LSTM, Bi-LSTM and Bi-GRU-LSTM-CNN;
- In Study 2, the results of the above DL models were compared and the different XAI methods were applied, such as SHAP and LIME, to validate the best DL model.

### 3.5. DL Algorithms

**Figure 3:**
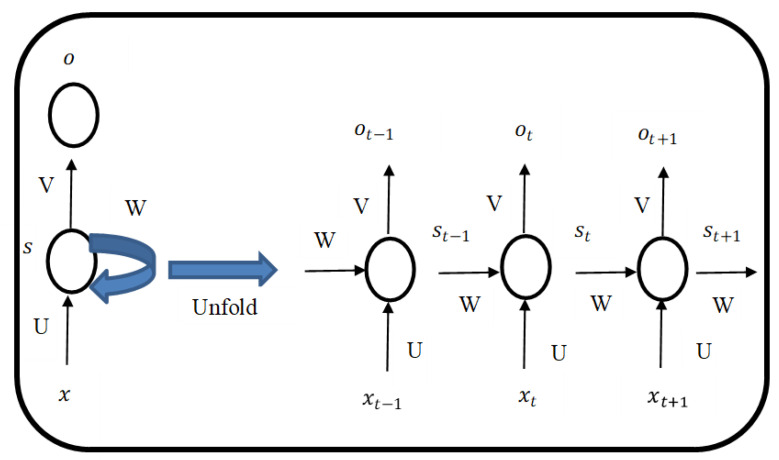
Showing RNN architecture.

A sequence of data input works well with an RNN [48]. In traditional neural networks, all the input variables are independent of the output variable. Some of the NLP problem examples, such as predicting if the sentence is positive or negative, spam classifier or time-series data, stock forecasting or sales forecasting, can be solved by RNN [49]. Bag of words, term frequency-inverse document frequency and Word2VEC are used for text preprocessing which convert text into vectors to solve NLP problems in machine learning. The issue with these algorithms is that they discard the sequence information in the sentence, thereby resulting in lower accuracy. The name “RNN” refers to the fact that each element in the sequence is subject to the same task, with the output being based on earlier calculations. It is expected that RNNs have memory, whereby they will keep track of data from earlier steps. However, in actual practise, they can only retrace a few steps [38]. Figure 3 shows a typical RNN architecture with respect to time-series data.

Assuming we have a sentence of five words, then the above figure will have five layers, with one layer for each word. In Figure 3, $x_{t}$ is the input, $s_{t}$ is the hidden state, and $o_{t}$ is the output step at time step *t*. The input at time step *t* is $s_{t}$ = f (U$x_{t}$ + W$s_{t-1}$). The function f is nonlinearity, such as Relu or tanh and $s_{t-1}$, which is required to initialize all elements to zeros when calculating the first state.

#### 3.5.1. LSTM and BiLSTM

**Figure 4:**
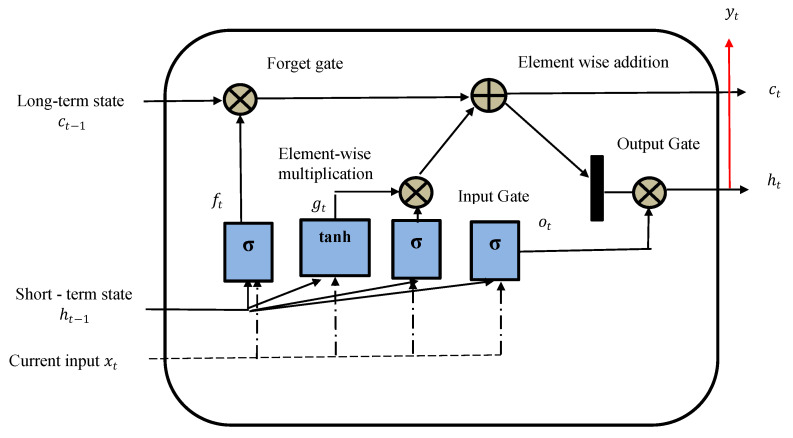
LSTM architecture.

LSTM is a gated RNN, and Bi-LSTM is an extension of the model. LSTM models can learn long dependencies from the previous states as compared to the traditional RNN model [50]. The Bi-LSTM model is an extension of the LSTM model, where it trains the input data twice using both forward and backward directions. Figure 4 shows the typical architecture of the LSTM model.

The forget and output gate manages the information that needs to be kept or deleted [51]. The model decision is jointly made by the LSTM block’s memory and the condition at the output gate. The output is then used as an input for the following step, creating a recurrent input sequence. The first model learns the input sequence, and the second model learns the reverse sequence (Figure 4). As we had two models trained in Bi-LSTM, we needed to combine them by using a merge step. Merging was performed using the following functions:

- Sum;
- Multiplication;
- Averaging;
- Concatenation (default).

#### 3.5.2. Bidirectional GRU

GRU, which was introduced in 2014, is similar to LSTM without an output gate. GRU has update and reset gates that help to combine new inputs with the previous ones [52]. The update gate decides how much previous memory needs be saved. In LSTM, the cell state and hidden state are known as short-term memory, whereas only one state, that is, the hidden state, is found in GRU. GRUs demonstrate better performance on smaller to medium quantity datasets.

#### 3.5.3. Developing DL Models

The results of the DL models were verified by adjusting the hyper parameters after multiple rounds of training and testing. The DL models were trained and tested several times before finalising the hyperparameters, which included epochs, batch size, layers, dropouts, number of units, and the activation function. The LSTM model was built with one embedding layer for word embedding, one spatialdropout1d layer to train fewer features, an LSTM layer, flatten layer, two dense layers with the second one using SoftMax, and one dropout layer with 50% located between the dense layers. One embedding layer for word embedding, one spatialdropout1d layer for training fewer features, one Bi-LSTM layer, a flatten layer, two dense layers with the second one using SoftMax, and one dropout layer with 50% located between the dense layers were used to create the Bi-LSTM model. The Bi-GRU-LSTM-CNN model was developed with one embedding layer for word embedding, one spatialdropout1d layer to train fewer features, one bi-directional GRU layer with two LSTMs (one forward and one backward), a 1D convolutional layer, one global average polling 1D later and one global max pooling 1D layer, two dense layers with the last one using SoftMax, and one dropout layer with 50% located between the dense layers.

The models achieved optimum results with 100 epochs and a batch size of 32 after testing various combinations of hyperparameters. The model was compiled with the Adam optimiser [53] and sparse categorical cross-entropy loss function [54]. All three classifiers included 80% data for training and 20% for testing the models. 

### 3.6. Assessment Measures

To understand the accuracy of the models, we used the confusion matrix and F1 score of precision and recall metrics of the ML and DL models. The formulae for calculating precision Equation (1) and recall Equation (2) are as follows:

(1) $$Precision=\frac{\begin{array}{c} True\;Positive \end{array}}{\begin{array}{c} True\;Positive+False\;Positive \end{array}},$$

(2) $$Recall=\frac{\begin{array}{c} True\;Positive \end{array}}{\begin{array}{c} True\;Positive+False\;Negative \end{array}}.$$

As can be seen from the above equations, precision should be used when the cost of false positives for the business is greater, whereas recall should be used when the cost of false negatives is greater for a business. The *F*1** score (Equation (3)) is used to seek a balance between the two metrics.

(3) $$F1=2\times \frac{\begin{array}{c} Precision\;*\; \end{array}Recall}{\begin{array}{c} Precision+Recall \end{array}}.$$

We used the *F*1** score to compare the accuracy of the DL models.

### 3.7. XAI Techniques

#### 3.7.1. SHAP

SHAP is a game theoretic method to explain ML models. It explains how to predict an instance *x* by computing each feature’s contribution to the prediction [55]. SHapley values are perturbation-based methods, where no hyperparameters are required, except for the baseline. The SHapley value Equation (4) is calculated as follows:

(4) $$Ri=\overset{}{\underset{S\subseteq P\backslash \left\{i\right\}}{\sum }}\frac{\left|S\right|!\left(\left|P\right|-\left|S\right|-1\right)!}{\left|P\right|!}\left[\hat{f}\left(S\;\cup \;\left\{i\right\}\right)-\hat{f}\left(S\right)\right],\;$$

where *P* represents a set of *N* players, and $\hat{f}$ maps each subset of *S* ⊆ *P* of players to real numbers. The result $\hat{f}$ (*P*) of the game is represented by the contributions of all players. The SHapley value for player *i* can be described as the average marginal contribution of player *I* to all possible combinations *S* that can be formed without it.

With $\hat{f}$ as the set function, the above equation can be implemented for neural network function *f*. We replaced$\;\hat{f}\;(S$) with $\hat{f}\;\left(x_{s}\right),$ where $x_{s}$ indicates the original input vector $x_{}$ with all features not present in *S* replaced by the baseline value. 

#### 3.7.2. LIME

LIME is a model-agnostic and concrete implementation of local surrogate models. LIME focuses on training the local surrogate models to explain individual predictions rather than the global surrogate model. LIME tweaks the feature value of a single data sample and checks for the change in the output. It also generates new texts by removing words randomly from the original text. 

A set of scores are produced by LIME, denoted as *E*, from a text classifier *C* and a text sequence *T*, where their elements indicate the relevance *r*(*t*) ∈ [−1, 1] of the word tokens (i.e., separated by white spaces) *t* ∈ *T* with respect to a given class *c* of interest [56]. LIME provides a positive score to tokens in *T* that push *C*’s prediction towards *c* and a negative score to tokens in *T* that push *C*’s prediction towards any other class *c*′ c. LIME provides a positive score to the tokens in *T* that push the prediction of *C* towards *c* and a negative score to those pushing to any other class *c*′ ≠ *c*.

## 4. Results

In this section, three DL techniques for sentiment analysis of the FDSs were evaluated. The accuracy of models was compared using the confusion matrix and model metrics. XAI techniques, such as SHAP and LIME, were used to explain the outcome of the DL models. 

### 4.1. Comparison of DL Models

**Figure 5:**
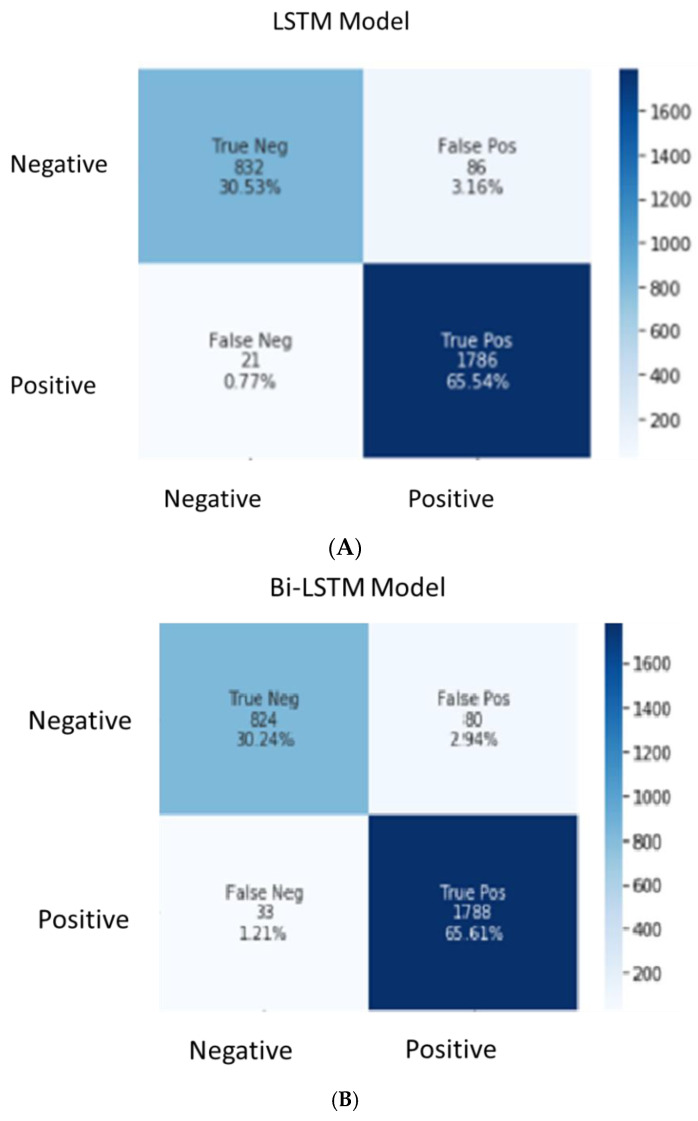

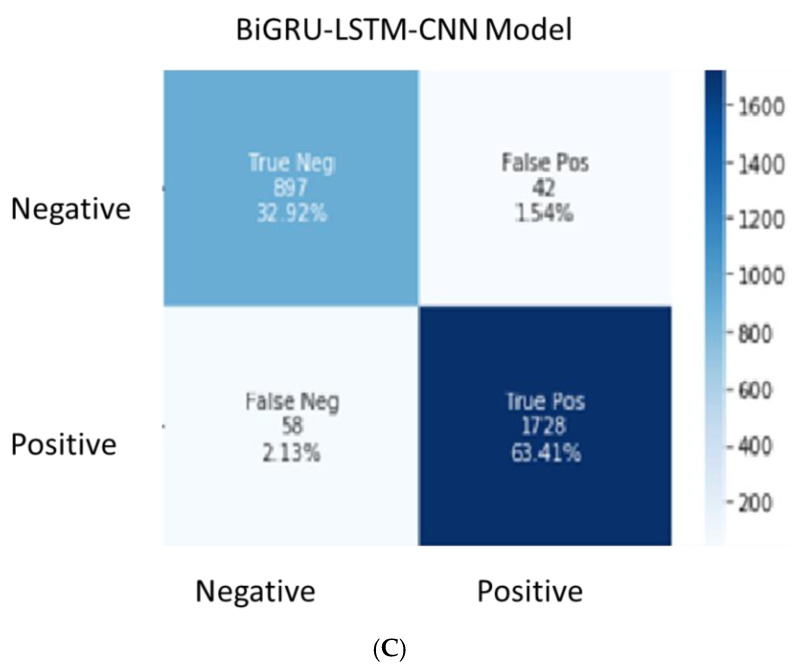
Confusion matrix of (**A**) LSTM; (**B**) Bi-LSTM; and (**C**) Bi-GRU-LSTM-CNN model.

The classification performance and errors of the classifiers are represented in the confusion matrix, as shown in Figure 5. The type 1 error is shown by false positives, and type 2 is shown by false negatives. The significance of an error is determined by the classification problem’s domain. In the case of FDS, higher importance is given to type 2 errors. A type 1 error occurs when an alert raised for a positive customer review is mistakenly detected as a complaint, which will require some operational effort to investigate and close the customer comment as not a complaint. A type 2 error indicates that the system cannot identify negative sentiments, which is a larger risk because the customer complaints will not be detected by the system. The FDS organisations prefer to identify and work on each and every customer complaint to improve customer satisfaction. Hence, the model should have fewer false negatives in its prediction.

The confusion matrix shown in Figure 5A clearly indicates that the LSTM classifier can perform accurate prediction (65.54% reviews, which are positive, and 30.53% reviews, which are negative), achieving an overall accuracy of 96.07%. Only 0.77% reviews give false negative results, whereas 3.16% return false positive results. The numbers from the confusion matrix were validated with the performance metrics (Table 2) by using the assessment measures. 

Similarly, the confusion matrix (Figure 5B) shows that the Bi-LSTM classifier can perform accurate prediction (65.61% reviews, which are positive, and 30.24% reviews, which are negative), resulting in a 95.85 percent overall accuracy. The Bi-LSTM classifier returns 1.21% false negative results, and 2.94% false-positive results. The numbers from the confusion matrix were validated with the performance metrics (Table 2) by using the assessment measures. 

The confusion matrix (Figure 5C) shows that the Bi-GRU-LSTM-CNN classifier can perform accurate prediction (63.41% reviews, which are positive, and 32.92%, reviews which are negative), resulting in a 96.33% overall accuracy. The Bi-GRU-LSTM-CNN classifier leads to 2.13% false negative results, and 1.54% false-positive results. The numbers from the confusion matrix were validated with the performance metrics (Table 2) by using the assessment measures.

The results from the above performance metrics show that all the DL models developed for performing a sentiment analysis attained a high overall accuracy (LSTM at 96.07%, Bi-LSTM at 95.85%, and Bi-GRU-LSTM-CNN at 96.33%). However, FDS organisations will pick the LSTM model as the best classifier due to its fewer type 1 errors with 21 false negatives as compared to BiLSTM with 33 and Bi-GRU-LSTM-CNN with 58. Although the accuracy of the LSTM model is high, it lacks model interpretability and explainability of the decisions made. The explanations of the LSTM-based black box model will help build trust in the system.

### 4.2. Explanation of LSTM Model Using XAI Techniques

This section describes the interpretation and explainability of the LSTM-based black box model by using different XAI techniques (SHAP and LIME). 

**Figure 6:**
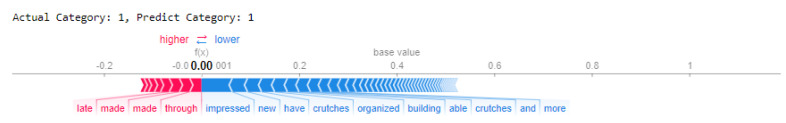
SHAP explanation of the positive customer review detected by the LSTM model.

**Figure 7:**
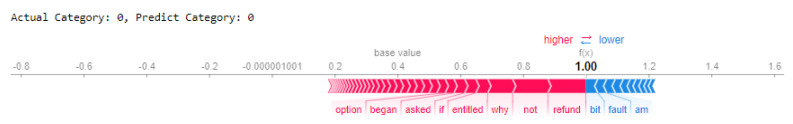
SHAP explanation of the negative customer review detected by the LSTM model.

We applied SHAP on the model to interpret the feature importance considered by LSTM while making the predictions after training and testing the LSTM classifier. A DeepExplainer class from the SHAP library, which took approximately 20 min, was used to generate the SHAP values for the test dataset. Figure 6 and Figure 7 show the force plot representing the interpretation of two customer review predictions made by the LSTM classifier. The base value shown on the plot is the average value of the target variable across the dataset we passed to the DeepExplainer class. Each arrow strip shows the effect of its associated feature on pushing the target variable away from or close to the base value. Red strips show that their associated feature pushes the value toward the higher side (indicating customer review being negative) in comparison to the base value, whereas the blue strips indicate that the associated feature pushes the value down on the lower side (indicating customer review being positive).

Figure 6 represents the SHAP explanation for the LSTM model’s detection of a positive customer review. The force plot and customer review suggest that the customer was very happy with the customer service received following their new delivery of the meal after requesting redelivery because the customer was on crutches. The words represented in blue contribute to positive sentiment, and the words shown in red contribute to negative sentiment. The explainable model demonstrated that words, such as ‘impressed’, ‘new’ and ‘have’, strongly pushed the output prediction value toward positive sentiment, which matches with the actual positive customer review prediction.

Figure 7 shows the SHAP explanation for negative customer review prediction. The inference from the force plot and customer review is that the customer is asking for a refund because the ordered subway came without salad and sauce. The words, such as ‘refund’, ‘not’, ’why’ and ‘entitled’, show a positive correlation with the negative customer review prediction.

As can be seen from Figure 6 and Figure 7, the LSTM model using the SHAP technique can validate whether the right words contribute to the right prediction. The SHAP interpretation identifies satisfactory reasoning for the predictions made by the LSTM model. It provides good insight for FDS organisations so that they can decide if the identified negative customer review is a false positive and whether it requires further investigation by inspecting these indicators along with the actual meaning of the customer reviews.

The LIMETextExplainer class from the LIME library was used to predict the class with variations in the probability value on the same two customer reviews previously used by SHAP. LIMETextExplainer took only 2–3 min to train and generate local explanations for predictions.

**Figure 8:**
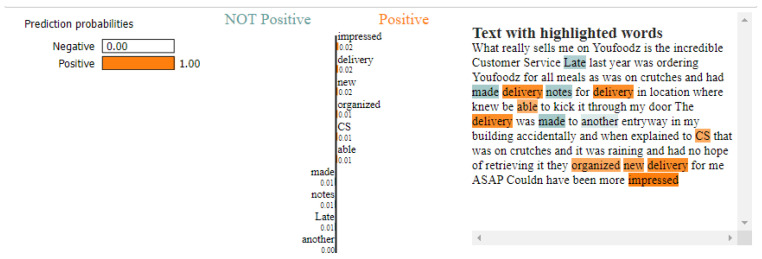
LIME explanation of the positive customer review detected by the LSTM model.

For customer review 1 in Figure 8, the LSTM model was 100% certain that the review indicated positive sentiment. The words, such as ’impressed’, ’delivery’ and ’new’, increased the review’s chance to be classified as positive. However, the feature contribution of the positive words classifying the customer review as positive looks similar in the LIME explainer graph.

**Figure 9:**
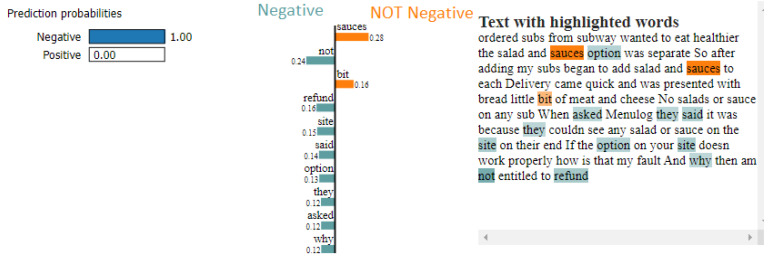
LIME explanation of the negative customer review detected by the LSTM model.

In the next example of customer review 2, the LSTM model was 100% certain that the customer review (shown in Figure 9) indicated negative sentiment. LIME explainer suggests that the words, such as ‘not’, ‘refund’ and ‘site’, show a positive correlation with the negative customer review prediction.

## 5. Discussion

In this study, we addressed the subject of sentiment analysis in the FDS domain by utilising DL approaches and interpreted them with XAI techniques. The study was able to solve the black-box nature of the DL methods [28] by implementing XAI techniques such as SHAP and LIME. This study experimented with the ProductReview website dataset of various FDS organisations, such as Menulog, Deliveroo, Uber Eats and Youfoodz across Australia, so as to cover all locations across Australia. This was one of the limitations identified by Luo [28] in their research work to test the robustness of the DL model across different restaurant locations. The research work’s main novelty and contribution was in developing a DL model to analyse customer sentiments in the FDS domain and explain such sentiments using the XAI technique to validate the model’s logic for industry use. FDS organisations can successfully use the data from complaints to discover areas for improvement in order to increase customer satisfaction. To the best of our knowledge, no investigation of the application of XAI techniques in DL methods in the FDS domain have been conducted as of yet. The recently published review paper presented a systematic review of DL and XAI in the FDS domain [13] and highlighted the importance of research work in the direction of DL along with XAI. 

DL techniques (LSTM, Bi-LSTM and Bi-GRU-LSTM-CNN) were used to perform a sentiment analysis of the customer reviews after performing data preprocessing. Table 2 shows that LSTM, Bi-LSTM and Bi-GRU-LSTM-CNN obtained an accuracy of 96.07%, 95.85% and 96.33%, respectively. The DL models achieved higher accuracy as compared to the models developed in the past in other research works. Table 3 shows the accuracy achieved in DL/Ml models when predicting customer sentiments in the FDS domain in recent papers. However, all the ML/DL methods used in the past were not interpretable. A recent study [28] found that DL models performed better than ML models. The Bi-LSTM model (92%) performed better than the Simple Embedding +Average Pooling model (90%), GBDT (88.9%) and Random Forest (86.6%). However, future work is recommended to explain the DL black boxes as they are non-interpretable. Additionally, work performed by [28] was carried out in a limited location dataset, hence the study recommended future work on a dataset with greater location coverage.

Compared with previous work in the literature represented in Table 3, we found that the DL models implemented in this research acquired higher accuracy. The research used the dataset from the ProductReview website which covers all the locations across Australia. The FDS organisations aim to identify and address each and every customer complaint without missing any to improve customer satisfaction. Thus, the model’s prediction should have fewer false negatives. Given that all the DL models achieved close accuracy levels, the model with fewer type 2 error (false negatives) was selected. To identify the best fit model for the FDS domain, the confusion matrix (Figure 5) was used to understand the false positive and false negative percentages in each of the DL models. Table 4 shows the details of false negative versus false positive results, along with the overall accuracy of the three DL models.

Although the overall accuracy of Bi-GRU-LSTM-CNN was 96.33%, its false negative percentage was 2.13, which was higher than that of the LSTM model (0.77). The overall accuracy of the LSTM model was 96.07%. In the case of sentiment analysis, the generation of fewer false negatives is preferred over false positives because businesses do not like to miss any negative customer reviews as compared to positive reviews. The LSTM model is recommended over the Bi-LSTM and Bi-GRU-LSTM-CNN models due to its lower false negative percentage. This model can be used to perform a sentiment analysis of any FDS organisation. The customer review data can be pulled from different social media channels, such as the ProductReview website, Twitter and Facebook. The negative customer reviews can be further analysed to understand which aspects of customer service can be improved. Positive customer reviews can be used by food delivery organisations for rewards and recognition.

Although the LSTM model achieved high accuracy in determining the sentiment of customer reviews, it did not provide explainability of the model. Placing trust in the highly accurate DL black-box model without knowing its decision-making logic is difficult for FDS organisations. Businesses prefer to verify the parameters or features of a model that provides accurate results. Following the training and testing of the LSTM classifier, we used SHAP and LIME techniques to interpret the feature importance considered by LSTM when making predictions for customer reviews. A DeepExplainer class from the SHAP library took more time to generate the SHAP values for the test dataset as compared to LIME.

SHAP and LIME provided similar prediction results with their explanation of the model (Figure 6, Figure 7, Figure 8 and Figure 9). The use of SHAP and LIME allowed us to perform an in-depth analysis of the model with its sample customer review test data. For positive customer reviews, SHAP and LIME picked key feature words, such as ‘impressed’, ‘new’, ‘delivery’ and ‘have’, which strongly pushed the output prediction value toward a positive sentiment, which matches with the actual positive customer review prediction. The feature contribution of the positive words classifying the customer review appears flat in the LIME explainer graph as compared to the SHAP force plot graph. Similarly, for negative customer sentiment review, the explainers suggested that words, such as ‘not’, ‘refund’, ‘why’ and ‘site’, show a positive correlation with negative customer-review prediction. However, SHAP took more time to train with the dataset compared to LIME. 

The strengths of this study depend on the LSTM model, which was effectively used to identify the positive and negative sentiments from customer reviews with an accuracy of 98.07% with a lesser false negative rate of 0.77%. The accuracy attained by the DL model was higher than the models used in past work in the FDS domain [28,29,30,31]. This study used the data from ProductReview, which is a popular website in Australia for sharing customer reviews on products and services. Further experiments demonstrated that XAI techniques, such as SHAP and LIME, can be used effectively on LSTM models to obtain the key features (words) contributing to the sentiment outcome. SHAP’s ability to reveal the interpretation of LSTM predictions by pinpointing the contribution score of each feature is better compared to LIME. This is also the first study in which SHAP and LIME were applied to an LSTM classifier in the FDS domain. 

The limitation of this research is that, in the presence of sarcasm, the model can misinterpret a customer review as positive and vice versa, thereby resulting in an improper training set. The risk of spam accounts, false accounts and bots, which can generate irrelevant data and affect the training set and compromise accuracy results, is always high.

Negative or positive sentiments can be predicted by using the LSTM model combined with XAI techniques with high accuracy and explainability. The recommendation for future work is to categorise the negative and positive sentiments into various topic groups so that they can be sent to the right channel to address supply chain issues. The negative sentiment topics can be used for customer satisfaction improvement, whereas the positive sentiment topics can be used to reward staff or restaurants.

## 6. Conclusions

The purpose of this study was to predict the tone of customer evaluations in the FDS industry and to explain the decisions. Even once the COVID-19 pandemic abates, the rise in FDS use will continue. Given the volume of review data dispersed across numerous platforms and the absence of customer service professionals to examine and respond to each comment, AI can help FDS organisations to solve problems and save money. In the FDS domain, a false positive result indicates more operational efforts, whereas a false negative increases the risk of an organisation missing important customer complaints. The results showed that the LSTM model with lower false negatives outperforms the BiLSTM and Bi-GRU-LSTM-CNN models. SHAP and LIME were successfully applied to the LSTM model to determine the positive or negative contributions of each word to the predictions made by the model. Original customer reviews were analysed, and the logic behind the predictions made by the DL models, such as LSTM, was found to be explainable. Therefore, this research revealed that the behaviour of such models can be understood by implementing DL models for sentiment analysis along with XAI techniques. The LIME explainer uncovered the features that contribute to a particular prediction, and the SHAP explainer can further deepen an organisation’s understanding of models’ behaviour. SHAP required more training time with the dataset compared with LIME. This research concludes that the sentiment analysis of customer reviews in FDS can be best achieved with the LSTM model combined with LIME and SHAP techniques to achieve high accuracy and explainability. In future research, the focus should be on topic categorisation techniques that can be added to DL with an XAI solution to direct customer complaints to the right problem-solving group so that they can address supply chain issues. Additionally, the same methodology can be used to predict customer reviews in other domains to compare the results. Furthermore, it is worth exploring the models in a real-time working scenario where, as soon as a customer lodges their complaint, the complaint is directed to the relevant department for resolution.

## Figures and Tables

**Table 1:** Different attributes of the dataset from ProductReview.

Username	Review Topic	Location	Star Ratings	Date	Review Comment
Mark A	Rubbish!	Sydney, NSW	1 star	3 February 2021	Hands down the worst delivery service, always slow, food or drinks missing, food cold... Won’t be using again, after waiting an hour for food I don’t want a voucher just to reorder.
Lucy	Refunding is a joke	Sydney, NSW	2 star	30 January 2021	Happy I got an actual refund instead of credit for once, but unhappy that they refused to refund me for ruined food caused by thoughtless and inappropriate packaging. Just use any other delivery app.
Les	When it actually works not a bad site to order food	Greater Melbourne	3 star	13 September 2021	This week all the Melbourne restaurants randomly drop off the site, and take some time to return. If you are unfortunate enough to have an order confirmed, it sits there unmoving until you call them and cancel it, so obviously it has no real recovery mechanism built into the software. Customer service people do their best but often they can only suggest cancelling the order and tying again later. Amateurish at best.
Lily	Good Service	Sydney, NSW	4 star	13 February 2021	Food was on time and hot, the ordering process was slightly confusing but other than that, it was great, good customer service and accurate tracking time! Definitely would use again.
Russell G.	First Time User	Sydney, NSW	5 star	20 January 2021	I provided a wrong address by accident. Driver called me up, advised how far away he was, met me at the door. Food is warm and well packaged-Happy with the Menulog service-will use again.

**Table 2:** Performance metrics-LSTM; Bi-LSTM; and Bi-GRU-LSTM-CNN model.

	DL Model	Precision	Recall	F1_score	OA
**Negative**	**LSTM**	0.98	0.91	0.94	96.07
**Positive**		0.95	0.99	0.97	
**Negative**	**BiLSTM**	0.96	0.91	0.94	95.85
**Positive**		0.96	0.98	0.97	
**Negative**	**BiGRULSTM**	0.94	0.96	0.95	96.33
**Positive**		0.98	0.97	0.97	

**Table 3:** Accuracy scores achieved in ML/DL models from recent papers.

Method	Accuracy	Interpretable	DL/ML	References
Random Forest	89%	No	ML	[28]
GBDT	87.5%	No	ML	[28]
Simple Embedding + Average Pooling	91.1%	No	DL	[28]
Bidirectional LSTM	90.8%	No	DL	[28]
SVM	91.5%	No	ML	[31]

**Table 4:** False negative vs. false positive.

	LSTM	Bi-LSTM	Bi-GRU-LSTM-CNN
False Negative	0.77	1.21	2.13
False Positive	3.16	2.94	1.54
Overall Accuracy	96.07	95.85	96.33

## Data Availability

The data presented in this study are available on request from the corresponding author. The data are not publicly available due to privacy.
